# Neurotransmitter and receptor systems in the subthalamic nucleus

**DOI:** 10.1007/s00429-023-02678-z

**Published:** 2023-07-21

**Authors:** Aron Emmi, Marta Campagnolo, Elena Stocco, Miryam Carecchio, Veronica Macchi, Angelo Antonini, Raffaele De Caro, Andrea Porzionato

**Affiliations:** 1 Institute of Human Anatomy, Department of Neuroscience, University of Padova, Padua, Italy; 2 Parkinson and Movement Disorders Unit, Centre for Rare Neurological Diseases, Department of Neuroscience, University of Padova, Padua, Italy; 3 Center for Neurodegenerative Disease Research (CESNE), University of Padova, Padua, Italy

**Keywords:** Subthalamic nucleus, Basal ganglia, Neurotransmitters, Dopamine, Serotonin, Parkinson’s disease

## Abstract

The Subthalamic Nucleus (STh) is a lens-shaped subcortical structure located ventrally to the thalamus, that despite being embryologically derived from the diencephalon, is functionally implicated in the basal ganglia circuits. Because of this strict structural and functional relationship with the circuits of the basal ganglia, the STh is a current target for deep brain stimulation, a neurosurgical procedure employed to alleviate symptoms in movement disorders, such as Parkinson’s disease and dystonia. However, despite the great relevance of this structure for both basal ganglia physiology and pathology, the neurochemical and molecular anatomy of the STh remains largely unknown. Few studies have specifically addressed the detection of neurotransmitter systems and their receptors within the structure, and even fewer have investigated their topographical distribution. Here, we have reviewed the scientific literature on neurotransmitters relevant in the STh function of rodents, non-human primates and humans including glutamate, GABA, dopamine, serotonin, noradrenaline with particular focus on their subcellular, cellular and topographical distribution. Inter-species differences were highlighted to provide a framework for further research priorities, particularly in humans.

## Introduction

The Subthalamic Nucleus (STh) is a lens-shaped subcortical structure located ventrally to the thalamus, that despite being embryologically derived from the diencephalon, is functionally implicated in the basal ganglia circuits. Because of this strict structural and functional relationship with the circuits of the basal ganglia, and in particular the motor circuits mediating movement suppression, the STh is a current target for deep brain stimulation (DBS), a neurosurgical procedure employed to alleviate symptoms in movement disorders, particularly Parkinson’s disease (PD) and dystonia (Temel et al. 2006; Antonini et al. 2018; Deuschl et al. 2022).

A prominent model of the STh’s functional anatomy is known as the tripartite model (Parent and Hazrati 1996; Joel and Weiner 1997; Keuken et al. 2012; Lambert et al. 2015; Alkemade and Forstmann 2014). According to this morpho-functional subdivision of the STh, the anterior STh is functionally related to the limbic circuit, the dorsolateral STh is involved in the motor circuit, and the ventromedial STh is involved in the associative circuit of the basal ganglia. However, the anatomical segregation between the subdivisions of the STh is debated, with several studies suggesting significant overlap between functional territories, especially in humans (Keuken et al. 2012; Alkemade and Forstmann 2015; Lambert et al. 2015). While animal models present inherent advantages over human studies, interspecies differences, particularly between rodents, non-human primates and humans, represent a crucial point in the ongoing debate on STh functionality (Hardman et al. 2002; Baunetz et al. 2011). This is particularly relevant since DBS is performed only in human subjects with specific clinical inclusion criteria (Antonini et al. 2018; Deuschl et al. 2022) and human tracer studies are limited to ex-vivo slow-diffusing dyes (Emmi et al. 2020). Indeed, while the rodent STh presents an open structure with most dendritic arborizations extending into other subcortical regions, in primates, and particularly humans, dendritic fields in the STh are confined to the anatomical boundaries of the nucleus (Rafols and Fox 1976; Alkemade and Forstmann 2014). Aside of structural differences, basal ganglia circuits in rodents and primates also appear to differ in terms of segregation of functional loops, as well as in the role played by different nuclei (Aoki et al. 2019; Joel and Weiner 1997; Emmi et al. 2020).

Recent evidence in rodents (Aoki et al. 2019) indicates segregation of limbic, associative and motor circuits, even though an unidirectional influence of the limbic over the motor loop via the substantia nigra pars reticulata (SNr) has been discovered. In non-human primates and humans, segregation seems to be maintained with regard to the associative loops (associative regions being contacted exclusively by other associative regions), but not at the level of the motor loops (motor regions being contacted by other functional divisions, such as the limbic and associative; Joel and Weiner 1997). Moreover, there appears to be a progressive shift from the substantia nigra pars reticulata (SNr) (in rodents) to the internal Globus Pallidus (GPi) (in primates) in mediating basal ganglia outputs to the thalamus, with subsequent consequences on subthalamo-pallidal and subtalamo-nigral projections (Hardman et al. 2002; Emmi et al. 2020). Indeed, Kelly and Strick (2004) did not find evidence of retrograde labeled axons in the SNr, but only in the GPi, upon STh tracer injection in non-human primates.

To further underline this aspect, tracer studies on the cortico-subthalamic tract (or hyperdirect pathway) in non-human primates performed by Haynes and Haber (2013) indicate both functional specificity and functional integration of limbic, associative and motor afferences to the STh. This refers to the topographical segregation of cortico-subthalamic projections to specific STh regions, paired with the notion derived from Rafols and Fox (1976)’s findings on wide-spanning dendritic arborizations in STh neurons. Hence, while STh afferences may present a precise regional topography (Haynes and Haber 2013), dendritic arborizations of STh may traverse multiple functional territories and receive information from more than one functional loop. Considering the increasing importance of the STh in processing different types of information through phylogenesis (Hardman et al. 2002), the definition of the STh’s role in open versus closed loop circuits could represent an important aspect regarding information processing within the human basal ganglia.

Nevertheless, despite evidence indicating functional intergation of STh afferences and the presence of functional gradients as opposed to functional territories with distinct boundaries, definition of “predominantly” limbic, associative and motor areas of the STh remains crucial for DBS in treating neurological and psychiatric diseases (Temel et al. 2000; Antonini et al. 2018; Deuschl et al. 2022). Indeed, while one one side there appears to be a progressive drive in supporting functional intergation between limbic, associative and motor functions in the STh, reflecting complex information processesing occurring in humans, on the other there is an urgent yet unmet need of defining safe-to-target regions of the STh during DBS, with the ultimate goal to reduce unwanted side effects while maximizing treatment effectiveness, improving patient quality of life (Rodriguez-Rojas et al. 2022).

This macro- and meso-scale level of investigation, with predominant focus on anatomical connections and broad functional gradients, requires further integration with the functional microscopic anatomy of the nucleus at a regional, cellular and even subcellular level.

Indeed, the hypothesized functional subdivision of the STh allows for the definition of functional specializations also at the cellular and subcellular level. As functional specialization of single cells is defined throughout development, neurons migrate and partially segregate according to their molecular profile, forming distinct populations with possibly distinct functions (Arendt 2008; Alkemade et al. 2019). This represents the framework upon which identification of specific neuronal populations, defined by distinct neurochemical markers, can lead to the identification of functional territories in the STh.

Methodologically, the neurochemical and receptorial organization of the STh has been investigated post-mortem through the aid morphological methods, such as in-situ RNA labeling techniques (in-situ hybridization, ISH, and fluorescent ISH, or FISH), immunohistochemistry, immunofluorescence and autoradiography, allowing the identification of neuronal subpopulations, and thereby potential functional subdivisions within the structure. Information on receptor expression and distribution within the STh could provide significant advantages in defining functionally segregated regions, thus advancing our understanding of structure’s subdivision and circuitry.

While we have previously described the morphology, topography and connectivity of the STh in humans and non-human primates (Emmi et al. 2020), recent work by Alkemade et al. (2019) has focused on the characterization of the functional microscopic anatomy of the structure, with particular regard to the distribution of GABAergic, glutamatergic, dopaminergic and serotoninergic signaling markers.

Despite this, the neurochemical and molecular anatomy of the STh, i.e. the expression and topographical distribution of neurotransmitters and receptor proteins within the structure, remains controversial and largely unknown (Alkemade et al. 2019). Few studies have specifically addressed the expression of receptors within the STh, and even fewer have investigated their topographical distribution within the nucleus. Furthermore, most data are derived from rodent and non-human primate studies, with little to no information being available on humans for the different systems of neurotransmitters and signaling molecules.

Finally, of the studies investingating the neurochemical anatomy of the STh in humans, very few consider the three-dimensionality of the structure or examine it in its whole rostro-caudal extent.

Hence, the aim of this review is to assess available studies in the literature addressing the expression and distribution of receptors, neurotransmitters and signaling molecules of relevance within the STh, differentiating between data deriving from animal studies (rodents and non-human primates) and findings in humans. The ultimate objective of this study is to further underline the need to validate and translate the findings deriving from animal models to humans, despite known methodological and technical limitations.

### Neurotransmitter systems and their receptors in the subthalamic nucleus

The following paragraphs describe the main neurotransmitter systems, the ionotropic and metabotropic receptors, as well as correlated structural and functional markers that are relevant for STh physiology and pathology. For each major system, a summary table and a schematic figure and is provided for quick orientation throughout included studies.

### Glutamatergic system

**Fig. 1:**
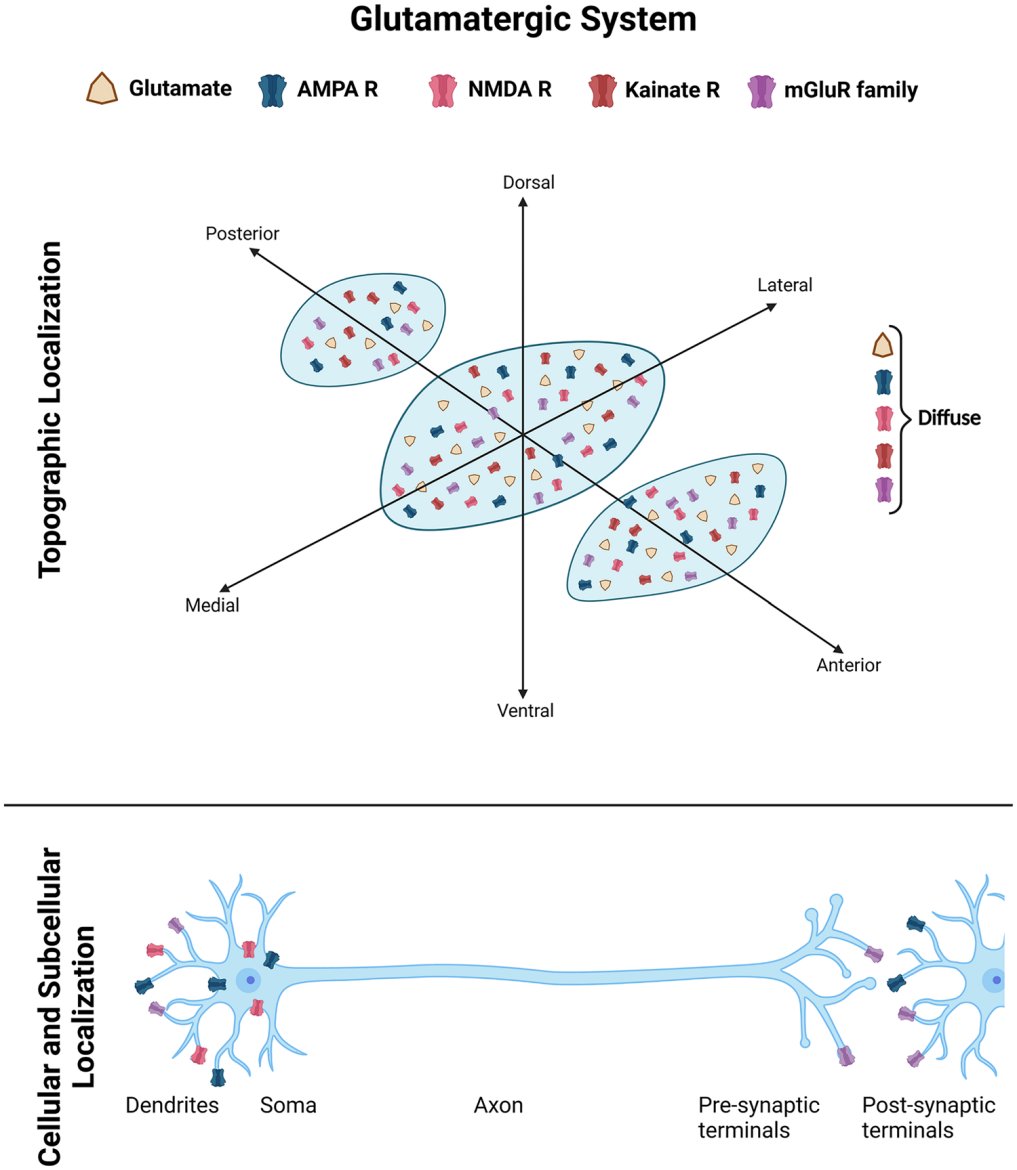
Glutamatergic system markers in the Subthalamic Nucleus (STh). Glutamate and its receptors represent the most characterized neurotransmitter system of the STh, with numerous studies addressing both its topography within the structure, as well as the cellular and subcellular localization of receptors. Graphical representations are based on the most recent available human and non-human primate studies, as reported in Table 1. In case of discordant findings, representation of marker topography was based on the most recent study addressing whole-volume distribution in the Subthalamic Nucleus

Glutamatergic system markers have been extensively investigated in rodents, non-human primates and humans, with little-to-no interspecies differences, suggesting that the glutamatergic system maintains its organization throughout phylogenesis. Studies addressing glutamatergic system markers are reported in Table 1. Figure 1 displays known topographical, cellular and subcellular localizations of glutamatergic system markers based on the examined studies.

**Table 1:** Glutamatergic system markers in the subthalamic nucleus

GLUTAMATErgic system					
Marker	Species	Method	Author	Expression	Topography
Glutamate	Non-human primate	Immunohistochemistry	Smith and Parent (1988)	Positive for STh Neurons	Diffuse distribution of glutamate positive neurons within the whole STh
	Rodent	Immunogold labeling and axonal tracing	Bevan et al. (1995)	Yes	Terminals (asymmetrical synapses) arising from the cortex and from the parafascicular nucleus of the thalamus
	Rodent	Immunogold labeling	Clarke et al. (1997)	Yes	Asymmetrical synapses
VGLUT1	Human	Immunohistochemistry, 3D reconstruction	Alkemade et al. (2019	Yes	Punctuate fiber labeling, boundaries of the structure
[3H]MK-801; [3H]glycine; [3H]CNQX; [3H]kainite (NMDA; AMPA and Kainate Receptor probes)	Human	Autoradiography	Ball et al. (1994)	Yes	Diffuse (whole STh)
AMPA Receptor and subunits (GluR1, GluR2, GluR3, GluR4)	Rodent	In situ hybridization	Sato et al. (1993)	Yes, except GluR3	Diffuse (whole STh)
(GluR1, GluR2/3)	Rodent	Immunohistochemistry	Petrialia and Wenthold (1992)	Yes	NS
(GluR1, GluR2/3/4c, GluR4)	Rodent	Immunohistochemistry	Martin et al. (1993)	Positive for GluR1, negative for GluR2/3/4c and GluR4	NS
(GluR1, GluR2/3, GluR4)	Rodent	Immunogold labeling	Clarke et al. (1997)	Positive for GluR1 and GluR2/3, negative for GluR4	Axodendritic synapses (asymmetrical synapses)
(GluR1 subunit, phosphorilated GluR1 and GluR2/3)	Non-human primate	Immunohistochemistry, Immunoelectronmicroscopy	Wang et al. (2000)	Prominent expression of GluR1	Postsynaptic; distal dendrites and soma
(GluR1, GluR2/3, GluR4)	Rodent	Immunofluorescence	Tai et al. (2001)	Yes	Diffuse (whole STh)
(GluR1)	Rodent	Immunohistochemistry	Lobo et al. (2003)	Yes	Diffuse (whole STh)
Kainate receptor and subunits (KA1, KA2, GluR5, GluR6, GluR7)	Rodent	Immunohistochemistry	Wullner et al. (1997)	Moderate for KA2, High for GluR6. Absent for other subunits	NS
(GluR5/6/7)	Rodent	Immunohistochemistry	Lobo et al. (2003)	Yes	Diffuse (whole STh)
(KA1, KA2, GluR5, GluR6)	Rodent	In-situ hybridization	Wüllner et al. (1997)	Yes, KA2	NS
NMDA Receptor (NMDAR subunit1)	Non-human primate	Immunohistochemistry, Immunoelectronmicroscopy	Wang et al. (2000)	Yes	Soma and dendrites
	Rodent	Immunohistochemistry	Lobo et al.(2003)	Yes	Diffuse (while STh)
(GluN2D subunit)	Rodent	Immunoelectronmicroscopy, Immunohistochemistry, Western blot	Swanger et al. (2015)	Yes	STh Dendrites
Metabotropic Glutamate Receptor Family (mGluR1)	Rodent	Immmunohistochemistry	Fotuhi et al. (1993)	Yes	NS
(mGluR2)	Human	Immunohistochemistry	Philips et al., (1999)	Yes	NS
(mGluR1; mGluR5)	Non-human primate	Immunohistochemistry, Immunoelectronmicroscopy	Wang et al. (2000)	Yes	Perisynaptic; distal dendrites
(mGluR1; mGluR2)	Non-human primate	Western immunoblot, Immunofluorescence	Kuwajima et al. (2004)	Yes	mGluR1, mGluR2 are located in close proximity to GABAergic synapses

Glutamatergic neurons represent the most conspicuous neuronal population in the STh. Glutamatergic STh neurons are considered type 1 projection neurons and are homogeneously distributed throughout the STh in rodents, non-human primates and humans (Iwahori 1978; Albin et al. 1989; Parent and Hazrati 1995; Shink et al. 1996; Clarke et al. 1997; Feeley Kearney and Albin 1999; Wang et al. 2000; Tai et al. 2001; Lévesque and Parent 2005). Similarly, glutamatergic fibers, whether afferent or efferent, have been detected throughout the whole structure across different species. In the rodents,glutamate was detected in correspondence to asymmetrical synapses (axo-dendritic synapses) (Bevan et al. 1995; Clarke et al. 1997), in particular at the level of synaptic terminals arising from the cortex and the parafascicular thalamic nucleus and directed towards the STh. In humans, Alkemade et al. (2019) detected vescicular glutamate transporter 1 (VGLUT1) immunoreactivity as punctuate fiber labeling, with increased density at the borders of the STh.

*Glutamatergic ionotropic receptors.* Glutamatergic ionotropic receptors (iGluR) comprise AMPA, NMDA, and Kainate receptor families. In the rodent STh, AMPA and NMDA receptors (and their subunits), are predominantly localized at the level of glutamatergic and gabaergic asymmetrical synapses (Clarke et al. 1997). Similarly, diffuse expression of GluR 5/6/7 subunits and binding for Kainate receptors was detected diffusely troughout the whole STh. (Wüllner et al. 1997; Lobo et al. 2003). In primates, Wang et al. (2000) evidenced that AMPA receptor subunit GluR1 is present in STh neurons, and is expressed at the level of the soma and proximal dendrites. GluR2/3 subunits were also detected, but were less expressed than GluR1. According to the authors, this indicates that some of the postsynaptic AMPA receptors may consist in homomers of GluR1. NMDA receptors were present in both soma and dendrites. According to Klockgether et al. 1991, AMPA receptor expression in STh is quantitatively predominant when compared to NMDA receptors, even though local application of NMDA antagonists in the STh decreases the metabolic activity of the STh neurons (Nakanishi et al. 1988; Blandini et al. 2001).

In the human STh, Ball et al. (1994) documented diffuse expression of NMDA, AMPA and Kainate receptors throughout the whole STh. Studies on the anatomical localization of iGluR in the STh do not suggest inter-species differences, nor indicate a defined topography within the structure. Nevertheless, no information concerning the subcellular localization of iGluR receptor subunits is available in humans, which have been extensively investigated in rodents and non-human primates.

*Glutamatergic metabotropic receptors.* Metabotropic glutamatergic receptors (mGluR) are diffusely distributed throughout the STh in rodents (Fotuhi et al. 1993), even though their cellular and subcellular localization were not investigated. In non-human primates, mild expression of mGluR subunits was detected at the level of the cell body, while more prominent expression was evidenced at the level of the distal dendrites (Kuwajima et al. 2004). In particular, Kuwajima et al. (2004) mGluR1 and mGluR5 were concentrated mainly in the synaptic area in close proximity to GABA-ergic synapses; Furthermore, while ionotropic glutamate receptors (AMPA and NMDA) were expressed mainly at a post-synaptic level, mGluR were located presynaptically (Wang et al. 2000).

In humans, Phillips et al. (1999) reported diffuse immunoreactivity to mGluR2 without specific topography; the subcellular localization of mGluR was not investigated in humans.

These findings suggest that, as for iGluR, mGluR are diffusely distributed throughout the STh with no relevant interspecies differences.

### GABAergic system

The STh receives prominent GABAergic afferences from the external segment of the Globus Pallidus (GPe) in rodents, non-human primates and humans (Smith and Parent 1988; Clarke et al. 1997; Emmi et al. 2020); In rodents and non-human primates, the majority of GABAergic pallidal terminals exhibited numerous varicosities, reminiscent of boutons en passant or boutons terminaux, forming synapses predominantly with proximal dendrites and less frequently with the soma and the distal dendrites (Parent and Hazrati 1995; Emmi et al. 2020). Other sources of GABAergic input to the STh, in rodents and non-human primates, include the pedunculopontine tegmental nucleus and the latero-dorsal tegmental nucleus (Usunoff et al. 2003). In humans, diffuse expression of GABA transporter 1 (GAT-1) documented by Augood et al. (1999) confirms that the STh is extensively innervated by GABAergic afferences. Recent evidence by Alkemade et al. (2019) confirmed moderate fiber terminal staining for Glutamic Acid Decarboxylase (GAD), which converts glutamate to GABA; presynaptic boutons were observed extending beyond the dorsolateral border of the STN, appearing as a cap on the dorsolateral tip of the nucleus.

While the presence of GABAergic terminals has been well established in all species, the presence of GABAergic neurons in the STh was initially controversial. Earlier studies in rodents (Yasumi et al. 1988) and non-human primates (Smith and Parent 1988) failed to identify GABAergic neurons in this structure, while later evidence identified GAD mRNA in STh cells across species (Levesque and Parent 2005), even though the neuronal nature of these cells, nor morphological features, were described. In humans, a study by Levesque and Parent (2005) evidenced that approximately 7% of the neurons within the human STh express GAD. The distribution of these neurons, which were morphologically identified as small Golgi type II interneurons, appears to follow an increasing dorsoventral gradient, with prominent density at the level of the ventral STh. According to the authors, the high density of GABAergic interneurons in the associative regions of the STh reflects the complex neural integration that underlies the anticipation, motivation and planning of movements which partakes within these territories. These findings are also supported by Alkemade et al. (2019) more recent study on humans. To date, no study to our knowledge has comparatively assessed GABAergic neurons across species, as previously performed by Hardman et al. (2002) for total neuronal populations. Hence, it is unclear whether the relative quantitiy, and topographic distribution, of GABAergic neurons in the STh differs across species.

### GABAergic receptors

Since the STh receives prominent GABAergic innervation from the globus pallidus, as previously stated, GABA-A and GABA-B receptors are widely distributed within the whole STh of rodents and non-human primates (Charara et al. 2000, 2004; Schwarzer et al. 2001; Galvan et al. 2004). GABA-A receptor subunits are expressed by small bi- and tripolar neurons within the rodent STh (Schwarzer et al. 2001); a similar expression of GABA-A receptor subunits within the non-human primate STh was reported Kultas-Ilinsky, Leontiev and Whiting (1998). GABA-B receptor subunits 1 and 2 are reported to be homogeneously distributed throughout the whole extent of the rodent and non-human primate STh (Charara et al. 2000, 2004; Galvan et al. 2004).

**Fig. 2:**
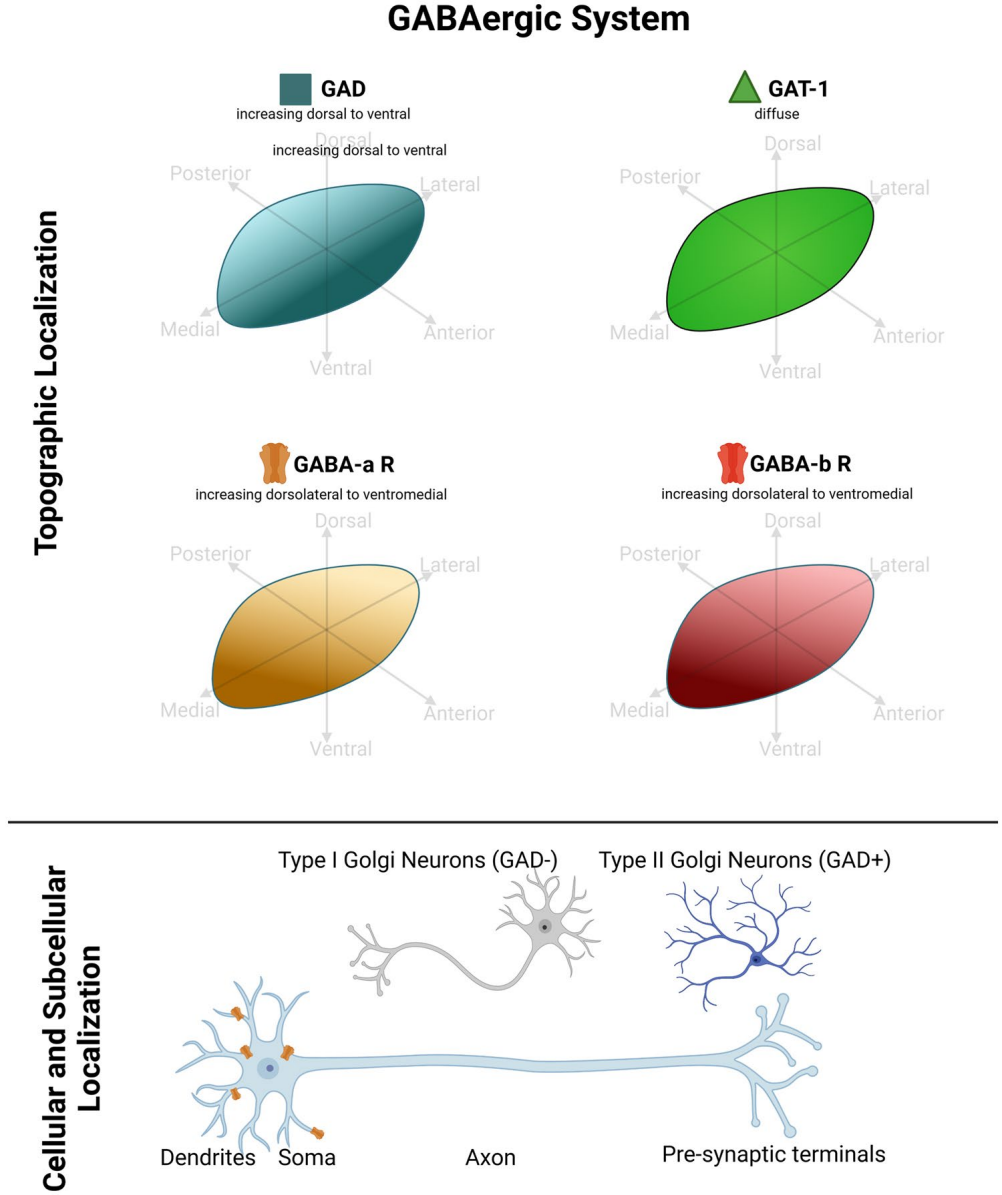
GABAergic system markers in the Subthalamic Nucleus (STh). GABAergic neurons (GAD +) represent a minor population of neurons within the STh, and are mainly located in the ventral aspects of the nucleus. An increasing dorsolateral to ventromedial gradient was found for the expression of GABA-a and GABA-b receptors. GABA-a receptors are found mainly on distal dendrites. Graphical representations are based on the most recent available human and non-human primate studies, as reported in Table 2. In case of discordant findings, representation of marker topography was based on the most recent study addressing whole-volume distribution in the Subthalamic Nucleus

In humans GABA-A receptor subunits have been described to follow a dorsolateral-ventromedial increasing gradient (Wu et al. 2018). The low immunoreactivity for GABA-A receptor subunits displayed by GAD-positive interneurons suggest that pallidal GABAergic projection neurons contact mainly glutamatergic projection neurons. Recent evidence by Alkemade et al. (2019) shows that GABA-A receptor subunit alpha 3 (GABRA3) is predominantly expressed in the neuronal soma, in addition to punctuate fiber staining.. Expression of GABA-B receptors in humans also appears to follow a similar dorsolateral-ventromedial increasing gradient of expression as GABA-A (Wu et al. 2018),

In the context of STh physiology, GABAergic afferences play a fundamental role in the modulation of the firing rate pattern of discharges and bursting activity. As seen above, GABAergic neurons in the STh do not appear to receive GABAergic afferences, which are mostly directed towards primary (glutamatergic) neurons. Furthermore, GAD + neurons do not express calcium binding proteins, such as parvalbumin, calbindin or calretinin, which are expressed in GAD− neurons. The increasing ventrolateral gradient of GABAergic interneurons evidenced by Lévesque and Parent (2005), coupled with the increasing dorsolateral to ventromedial gradient of expression of GABA-A and GABA-B receptors on primary neurons described by Wu et al. (2018), could reflect the functional specificity of this region, as associative areas generally contain more interneurons for the fine-tuning of the incoming signals. Nevertheless, the presence of topographically defined populations of GABAergic neurons, as well as the expression patterns of GABA receptors requires further investigation in non-human primates and rodents. In fact, it is yet unclear wheter GABAergic neuronal populations in the STh increase throughout phylogenesis, and if this phenomenon is limited to the ventromedial aspects of the nucleus, as seen in humans. Interspecies studies could provide useful information concerning the development of the STh throughout phylogenesis, clarifying whether local GABAergic interneuronal populations are related to the shift from segregated towards integrated circuits in the basal ganglia. Studies addressing GABAergic system markers are reported in Table 2. Figure 2 displays known topographical, cellular and subcellular localizations of GABAergic system markers based on the examined studies.

**Table 2:** GABAergic system markers in the subthalamic nucleus

GABAergic System					
Marker	Species	Method	Author	Expression	Topography
GABA	Non-human primate	Immunohistochemistry	Smith and Parent (1988)	Positive for terminals within the STh, but not STh neurons	NS
	Rodent	Immunogold labeling	Clarke et al. (1997	Yes	Symmetrical synapses deriving from the GPe
GAT-1 transporter	Rodent	In situ Hybridization	Yasumi et al. (1997)	Yes	Diffuse and intense positivity
	Human	In situ Hybridization	Augood et al. (1999	Yes	Diffuse
GABA-A Receptor and subunits	Non-human primate	In situ Hybridization, Autoradiography	Kultas-Ilinsky et al. (1998)	High (α2, β2, γ2 subunits) and Moderate (α3, β3,δ subunits)	NS
	Rodent	Immunohistochemistry	Schwartzer et al. (2001)	Yes	Small bi- and tripolar neurons within the STh
	Human	Immunohistochemistry	Wu et al. (2018)	Yes	Increasing gradient of immunoreactivity from the dorsolateral to the ventromedial aspect
GABA-A Receptor subunit 3	Human	Immunohistochemistry, 3D Reconstruction	Alkemade et al. (2019)	Yes	Neuronal staining and punctuate fiber staining
GABA-B Receptor and subunits	Non-human primate	Immunohistochemistry	Charara et al. (2000)	Yes	Diffuse
GABA-B Receptor1 subunit	Non-human primate	Immunohistochemistry, Immunoblot	“ ”	Yes	Diffuse
GABA-B Receptor2 subunit	Non-human primate	Immunohistochemistry	Charara et al. (2004)	Yes	Diffuse
GABA-B Receptor subunits	Non-human primate	Immunohistochemistry, Immunogold labeling	Galvan et al. (2004)	Yes	Diffuse
	Human	Immunohistochemistry	Wu et al. (2018)	Yes	Increasing gradient of immunoreactivity from the dorsolateral to the ventromedial aspect
Glutamate-decarboxylase (GAD)	Human	Immunohistochemistry	Nisbet et al. (1996)	Yes	NS
	Rodent	In situ Hybridization	Yasumi et al. (1997)	No	
	Human	Immunohistochemistry, Stereology	Levesque and Parent (2005)	Yes	Increasing dorsoventral gradient of immunoreactivity
	Human	Immunohistochemistry	Wu et al. (2018)	Yes	NS for GAD
	Human	Immunohistochemistry, 3D Reconstruction	Alkemade et al. (2019)	Yes	Fiber terminal staining, occasional reactive neurons

### Dopaminergic system

Dopaminergic afferences to the STh derive from the substantia nigra pars compacta (SNpc) (Emmi et al. 2020), even though the extent of dopaminergic innervation of the STh remains to be elucidated. In rodents, a conspicuous dopaminergic nigro-subthalamic bundle has been described (Hassani et al. 1997). Non-human primate studies demonstrated that labeled axons originating from the mediodorsal part of area A9 terminated mainly in the anteromedial STh, whereas those originating from area A8 terminated diffusely throghout the STh (François et al. 2000), suggesting a specific arrangement of dopaminergic fibers originating from the SNpc. Human studies, on the other hand, suggest prominent dopaminergic innervation of non-motor regions of the STh, particularly the anterior and ventromedial pole (Hedreen et al. 1999; Emmi et al. 2022). Diffuse projections throughout the whole STh are also reported in humans (Augood et al. 2000). Hedreen et al. (1999) evidenced that dopaminergic fibers present fine axonal branching patterns, compatible with terminal arborizations, only in the non-motor (containing calretinin + neurons) regions of the STh, while larger non-terminal axons could be detected in the motor areas. These findings suggest that dopaminergic innervation occurs at the level of the non-motor regions of the STh, while dopaminergic fibers travel across motor areas of the STh, likely directed towards the striatum, without forming synapses (Hedreen et al. 1999).

### Dopaminergic receptors

Similar to dopaminergic innervation of the STh, the expression of dopaminergic receptors remains controversial and poorly understood, particularly in humans. The most studied receptors include dopaminergic receptor D_1_ and D_2,_, with few studies investigating other dopaminergic receptor families. In rodents, dopaminergic receptor D_1_ displayed variable immunoreactivity that ranged from absent to moderate across studies (Dawson et al. 1986; Dubois et al. 1986; Fremeau et al. 1991; Johnson et al. 1994). Only Savasta et al. (1986) reported a high expression of D_1_R whithin rodent STh, while Mansour et al. (1992) evidenced dense D_1_ receptor binding but no evidence of D_1_ mRNA. This suggest that while D_1_R proteins may be present, STh neurons do not express D_1_R mRNA; this uncoupling between protein immunoreactivity and mRNA expression generally suggests D_1_R protein expression in afferent axons targeting the STh, with the corresponding cell bodies expressing D_1_R mRNA being located elsewhere. Indeed, non-human primate studies detected presynaptic D_1_R on preterminal axons of putative glutamatergic and GABAergic terminals (Galvan et al 2014). Studies of the human STh reported no evidence for D_1_R expression (Augood et al. 2000; Hurd et al. 2001) D_2_R expression in rodent STh was reported as low by Dubois et al. (1986) and moderate by Johnson et al. (1994), without specific topogaphy. In non-human primates, D_2_ receptors were found presynaptically, on preterminal axons and putative glutamatergic and GABAergic terminals, similarly to what has been described for D_1_R(Galvan et al. 2014).

Studies on humans reported conflicting results as far as D_2_R is concerned, ranging from negative (Augood et al. 2000), to low (Hurd et al. 2001) or moderate (Wang et al. 2000), with little-to-no topographic information available. In our recent studies on the human STh, D_2_R was expressed predominantly at the level of the dendritic spines of β-III-tubulin positive neurites with a decreasing ventral to dorsal gradient (Emmi et al. 2022). Non-neuronal expression of D_2_R was also found, suggesting astrocytic expression of D_2_R. The expression of other dopaminergic receptors, such as D_3_R and D_4_R, was documented in humans by Wang et al. (2000) and Matsumoto et al. (2002) respectively, but their topographical distribution was not reported.

**Fig. 3:**
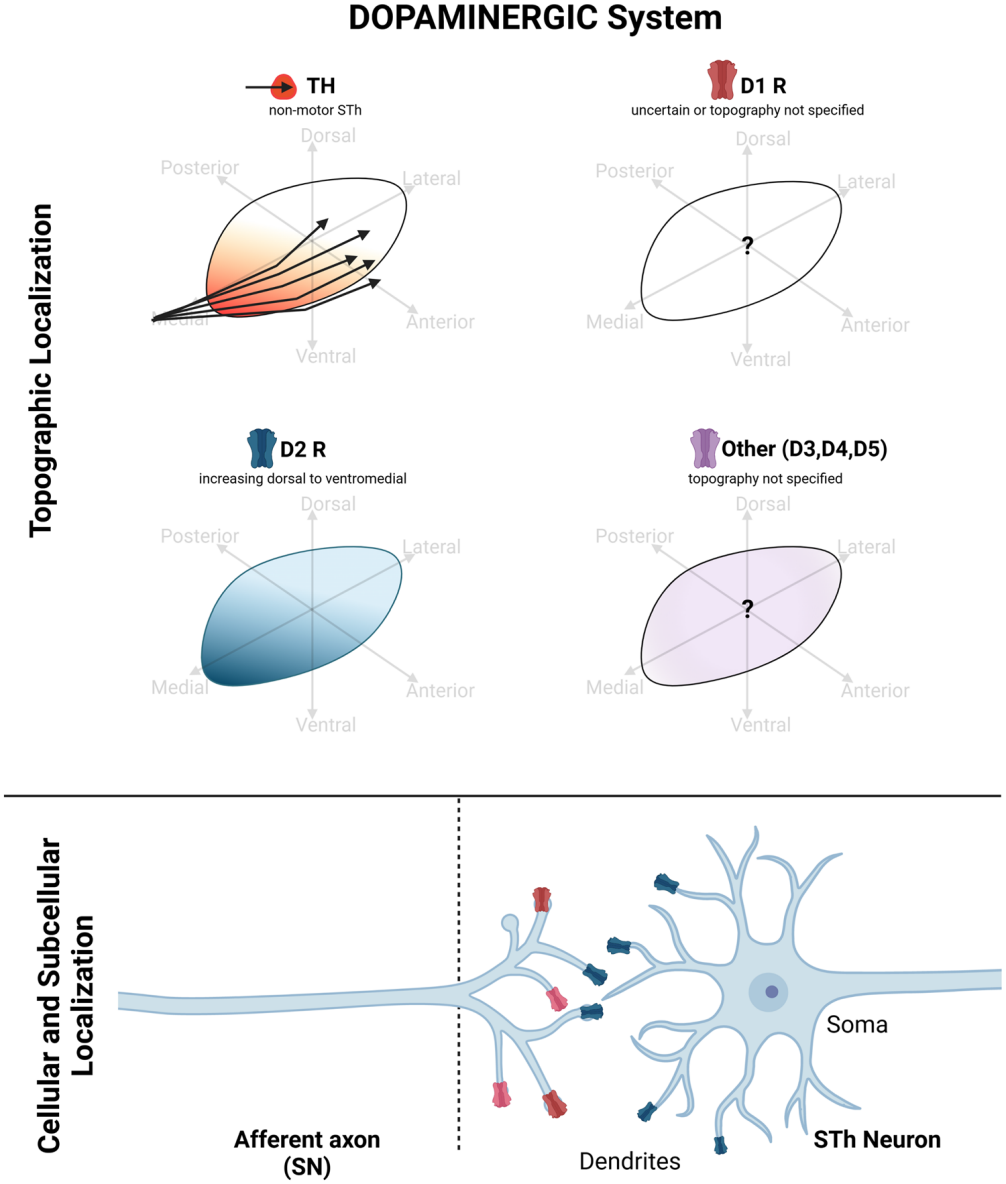
Dopaminergic system markers in the Subthalamic Nucleus (STh). Tyrosine Hydroxylase (TH) + fibers, deriving from the dopaminergic substantia nigra, are known to course ventrally to the STh and through the structure at the level of the ventromedial pole. The expression of dopaminergic receptors, in particular D1, D3, D4 and D5 is poorly known in humans. D2 receptors are found in a dorsolateral to ventromedial increasing gradient at the level of preterminal synapses and dendritic spines. Graphical representations are based on the most recent available human and non-human primate studies, as reported in Table 3. In case of discordant findings, representation of marker topography was based on the most recent study addressing whole-volume distribution in the Subthalamic Nucleus

Several aspects concerning the dopaminergic system in the STh remain to be investigated. In particular, the extent and exact topography of dopaminergic afferences, and whether or not these present terminal arborizations rather than passing fibers, remains to be elucidated. This is particularly relevant, as small dopaminergic terminal axons may modulate the activity of the STh, with particular regard to the non-motor regions, which appear to be more prominently interested by this (Hedreen et al. 1999); the consequence of the dopaminergic deafferentation of the STh in PD is poorly understood, but it could participate in the hyperactivity of the STN observed in animal models of Parkinson's disease. Indeed, it has been demonstrated that the increased activity of STh neurons following midbrain dopaminergic lesion cannot be due solely to removal of pallido-subthalamic inhibition, and it has been suggested that the intrinsic dopaminergic innervation of the STN could also participate in its hyperactivity (François et al. 2000). Furthermore, the exact subcellular localization of dopaminergic receptors, whether pre- or post-synaptically, remains to be elucidated in humans, and may represent another relevant factor in determining STh alterations following dopaminergic denervation. Studies addressing dopaminergic system markers are reported in Table 3. Figure 3 displays known topographical, cellular and subcellular localizations of dopaminergic system markers based on the examined studies.

**Table 3:** Dopaminergic system markers in the subthalamic nucleus

DOPAMINERGIC system					
Marker	Species	Method	Author	Expression	Topography
[3H]Mazindol (Dopamine uptake probe)	Rodent	Autoradiography	Javitch et al. (1985)	High	
Tyrosine Hydroxylase	Human	Immunohistochemistry	Hedreen et al. (1999)	Yes	Nonmotor regions of the STh, in correspondence to regions containing calretinin positive neurons
	Human	Immunohistochemistry	Prensa et al. (2000)	Yes	Fibers coursing dorsolaterally to the STh, giving off small collaterals to the STh
	Non-human primates, Humans	Immunohistochemistry	François et al. (2000)	Yes	Fibers originating from area A9 terminated in the anteromedial STh, while fibers from area A8 terminated diffusely throhgout the STh
	Human	In situ Hybridization + Immunohistochemistry, Autoradiography	Augood et al. (2000)	Yes	Diffuse
	Human	Immunohistochemistry, 3D reconstruction	Alkemade et al. (2019)	Yes	Thick and long, as well as thin punctate fibers
	Human	Immunohistochemistry	Emmi et al. (2022)	Yes	Coursing ventrally to the nucleus, and entering from the ventromedial pole
Dopamine receptor 1 (D1)	Rodent	Autoradiography	Dubois et al. (1984)	Moderate	
	Rodent	Autoradiography	Johnson et al. (1994)	No	
	Rodent	Autoradiography	Dawson et al. (1986)	Mild to moderate	
	Rodent	Autoradiography	Savasta et al. (1986)	High	
	Rodent	In situ Hybridization + Autoradiography	Fremeau et al. (1991)	No	
	Rodent	In situ Hybridization + Autoradiography	Mansour et al. (1992)	Uncertain	Dense receptor binding, no D1 mRNA
	Human	In situ Hybridization + Immunohistochemistry, Autoradiography	Augood et al. (2000)	No	
	Human	In situ Hybridization	Hurd et al. (2001)	No	
	Monkey	Immunoelectronmicroscopy	Galvan et al. (2014)	Mild	Preterminal axons
Dopamine receptor 2 (D2)	Rodent	Autoradiography	Dubois et al. (1984)	Mild	
	Rodent	Autoradiography	Johnson et al. (1994)	Moderate	
	Human	In situ Hybridization + Immunohistochemistry, Autoradiography	Augood et al. (2000)	No	
	Human	In situ Hybridization + immunohistochemistry	Wang et al. (2001)	Moderate	NS
	Human	In situ Hybridization	Hurd et al. (2001)	Mild	
	Non-human primate	Immunoelectronmicroscopy	Galvan et al. (2014)	Mild	Preterminal axons
	Human	Immunohistochemistry	Emmi et al. (2022)	Mild to Moderate	Dendritic localization, ventromedial-to-dorsal decreasing gradient of expression
Dopamine receptor 3 (D3)	Human	In situ Hybridization + Immunohistochemistry	Wang et al. (2000)	Yes	NS
Dopamine receptor 4 (D4)	Human	In situ Hybridization	Matsumoto et al. (1996)	Yes	NS
Dopamine receptor 5 (D5)	Non-human primate	Immunoelectronmicroscopy	Galvan et al. (2014)	Mild	Preterminal axons

### Serotoninergic system (5-HT)

**Fig. 4:**
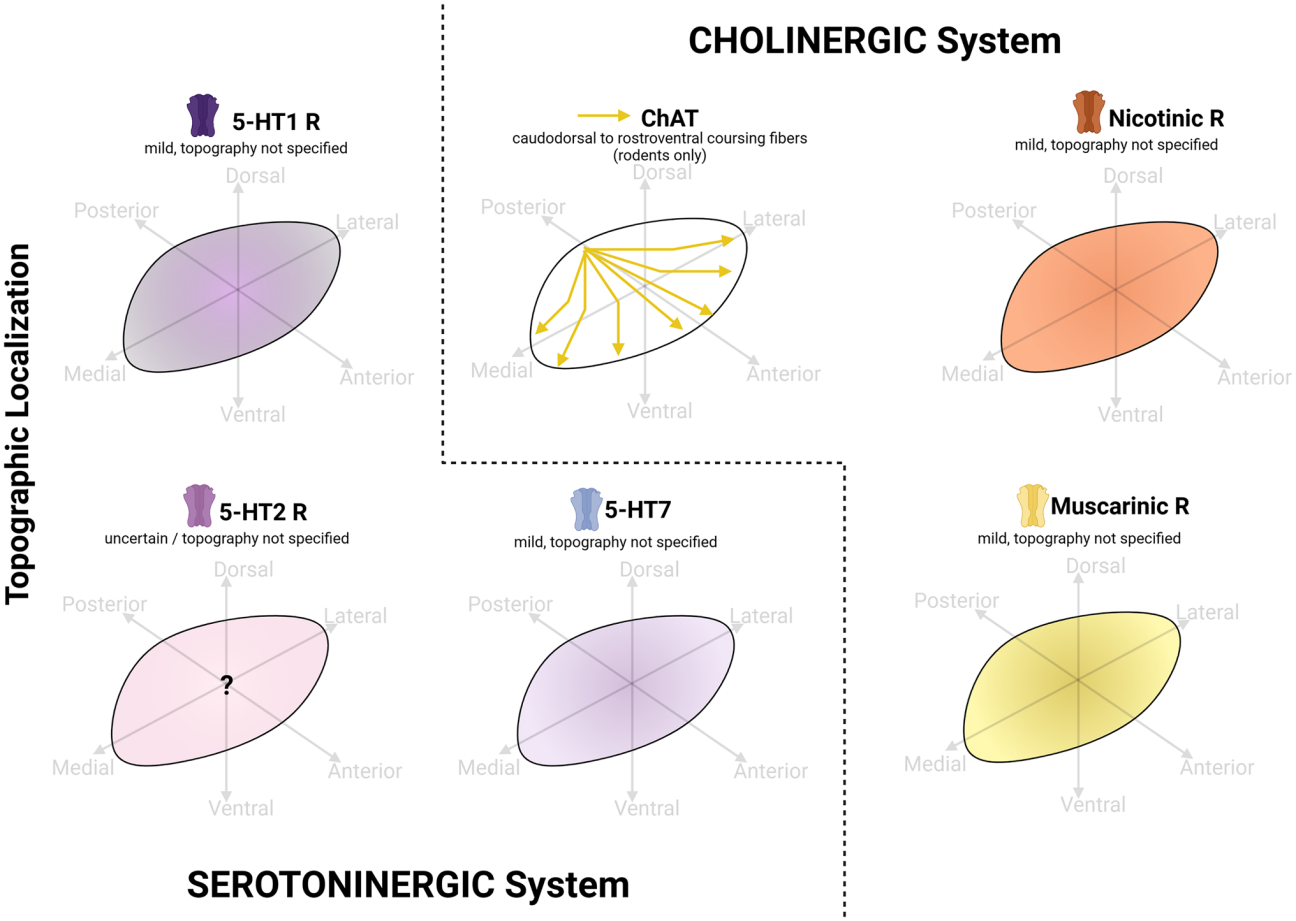
Serotoninergic and Cholinergic system markers in the Subthalamic Nucleus (STh). Serotonin receptors families were mainly investigated in rodents and non-human primates, and no topographical organization was reported in literature. The course of cholinergic fibers throughout the STh was investigated in rodents, and requires confirmation in humans. Nicotinic and muscarinic receptor expression was reported, but not their topography. Graphical representations are based on the most recent available human and non-human primate studies, as reported in Tables 4 and 5. In case of discordant findings, representation of marker topography was based on the most recent study addressing whole-volume distribution in the Subthalamic Nucleus (Table 6)

Serotoninergic (5-HT) innervation of the STh has been widely discussed in rodents, non-human primates and humans (Parent et al. 2011; Emmi et al., 2020). Across species, and particularly evident in non-human primates and humans, serotoninergic axons targeting the STh mostly derive from a single bundle of axons detaching from the main serotoninergic pathway, coursing in the lateral hypothalamic area and following the track of the lenticular fasciculus along the dorsal surface of the STh. A second smaller bundle is known to run along the ventral surface of the STh (Parent et al. 2011). In rodents, an increasing density of 5-HT immunoreactive fibers in the caudal part of the nucleus was detected (Steinbush et al. 1981), while also numerous terminal fibers were identified in the medial and ventral aspects of the STh (Mori et al. 1985). In non-human primates and humans, this is confirmed to occur particularly in the anteromedial and anterior-ventral aspects of the STh (Mori et al. 1985; Parent et al. 2011). Serotonin Transporter (SERT) positive fibers were described throughout the structure by both Martín-Cora and Pazos (2004) and Alkemade et al. 2019 in humans. Studies addressing 5-HT system markers are reported in Table 4. Figure 4 displays known topographical, cellular and subcellular localizations of 5-HT system markers based on the examined studies.

**Table 4:** Serotoninergic system markers in the Subthalamic Nucleus

5-HT System					
Marker	Species	Method	Author	Expression	Topography
SERT	Human	Immunohistochemistry, 3D Reconstruction	Alkemade et al. (2019)	Yes	Fibers within the STh
5-HT1 receptors	Human, Non-human primates, Rodents	Autoradiography	Waeber et al. (1989)	Yes	NS
5-HT1a	Rodent	In situ hybridization, immunohistochemistry, autoradiography	Reznitsky et al. (2016)	No mRNA nor Binding sites	
5-HT1b	Rodent	In situ Hybridization	Marteaux et al. (1992)	Yes	Diffuse Mild expression
	Rodent	Autoradiography	Brunivels et al. (1993)	Yes	Moderate expression
	Rodent	In situ hybridization, autoradiography	Boschert et al. (1994	Yes, both mRNA and binding sites	
	Rodent	In situ hybridization, immunohistochemistry, autoradiography	Reznitsky et al. (2016)	Yes	
5-HT1c	Rodent	In situ hybridization	Wright et al. (1995)	Yes	
5-HT1d	Rodent	Autoradiography	Brunivels et al.(1993)	Yes	Diffuse Mild expression
5-HT2 receptors					
5-HT2a	Rodent	In situ hybridization	Pompeiano et al. (1994)	No	
	Rodent	In situ hybridization, immunohistochemistry, autoradiography	Reznitsky et al. (2016	No mRNA nor Binding sites	
5-HT2c	Rodent	In situ hybridization	Pompeiano et al. (1994)	Yes, high levels of mRNA	
	Rodent	In situ hybridization	Eberle-Wang et al. (1997	Yes, high levels of mRNA	
	Rodent	Immunohistochemistry	Clemett et al. (2000)	Yes	
	Non-human primate	In situ hybridization	Lopez-Giménez et al. (2001	Yes	
	Rodent	In situ hybridization, immunohistochemistry, autoradiography	Reznitsky et al. (2016)	Yes	
5-HT4 receptor	Rodent	In situ hybridization, autoradiography	Vilaro et al. (2005)	Yes, both mRNA and binding sites	
5-HT7 receptor	Human	Autoradiography	Martin-cora et al. (2004)	Yes, moderate binding

### 5-HT Receptors

The expression of 5-HT receptors within the rodent STh has been widely explored in literature, and also documented in primates and humans (Waeber et al. 1989). Reznitsky, et al. (2016)’s study did not detect 5-HT1a receptor mRNA and binding sites, while several studies reported the expression of 5-HT1b receptor in rodents (Bruinvels, Palacios and Hoyer 1993; Boschert et al. 1994; Reznitsky, Plenge and Hay-Schmidt 2016). Rodent STh was also reactive for 5-HT1c (Wright et al. 1995) and 5-HT1d receptors, the latter displaying a diffuse distribution and mild immunoreactivtiy (Bruinvels, Palacios and Hoyer 1993). Concerning 5-HT2 receptors, rodent STh was negative for 5-HT2a (Pompeiano, et al. 1994; Reznitsky et al. 2016), but positive for 5-HT2c (Pompeiano, et al. 1994; Eberle-Wang et al. 1997; Clemett et al. 2000; Reznitsky, et al. 2016). In particular, Pompeiano et al. (1994) and Eberle-Wang et al. (1997) evidenced high levels of 5-HT2c mRNA. 5-HT2c has also been detected in primates (López-Giménez et al. 2001). The STh was also positive for mRNA and binding sites for 5-HT4 in rodents (Vilaró, et al. 2005). Concerning the human STh, moderate binding for 5-HT7 receptor was detected thorugh autoradiography (Martín-Cora and Pazos 2004). Hence, while an anterio-medial and antero-ventral 5-HT innervation has been described across species, particularly evident in primates, the topography of 5-HT receptor families within the structure remains to be investigated, as the majority of studies available in literature have focused on rodents.

### Cholinergic system (acetylcholine)

The cholinergic innervation of the STh appears to be uncertain and controversial. Even though the STh receives afferences from the pedunculopontine tegmental nucleus, which was believed to be an exclusively cholinergic nucleus, the recent discovery of consistent glutammatergic and dopaminergic neuronal populations within the PPT has questioned whether these connections are cholinergic or glutammatergic in nature (Marani et al. 2008). Choline acetyltransferase (ChAT) was detected in fibers coursing through the STh in rodents (Woolf and Butcher, 1986; Clarke et al. 1997; Kita and Kita 2011). These fibers originate from the peduncolopontine nucleus (Woolf and Butcher 1986) and travel through the STh from the caudodorsal to the rostroventral surface, giving off thin branches with small boutons (Kita and Kita 2011). The terminals of these fibers were enriched with glutamate, but not with GABA (Clarke et al. 1997). Studies on the porcine STh indicate prominent cholinergic innervation (Larsen et al. 2004). In humans, ChAT-rich thick and straight axons are reported to enter the STh dorsally and densely innervate the structure with rich terminal arborizations (Mesulam et al. 1992). Very finely varicose axons seemed to encircle subthalamic neurons, giving the appearance of a honeycomb pattern (Mesulam et al. 1992). The topography of these fibers, however, has not been investigated throughout the rostro-caudal extent of the structure.

*Nicotinic and muscarinic receptors.* In non-human primates, mild expression of nicotinic receptor α3, α4 and α7 subunits was reported, while β2 and β4 nicotinic receptor subunit expression was uncertain (Cimino et al. 1992; Quik et al. 2000). The non-human primate STh was not reactive for α6 and β3 subunits. Nicotinic receptors have been evidenced also in humans through autoradiography (Pimlott et al. 2004), and a moderate expression of α4 subunit mRNA was identified by Agulhon et al. (1998) within the fetal human STh. Low expression of muscarinic M2 receptors in the human STh was documented by Warren et al. (2007).Studies addressing cholinergic system markers are reported in Table 5. Figure 4 displays known topographical, cellular and subcellular localizations of cholinergic system markers based on the examined studies.

**Table 5:** Cholinergic system markers in the subthalamic nucleus

Cholinergic System					
Marker	Species	Method	Author	Expression	Topography
ChAT	Rodent	Immunohistochemistry, tracing methods	Woolf and Butcher (1986)	Yes, numerous fibers coursing through the structure	Originating from the pedunculopontine nucleus
	Rodent	Immunohistochemistry	Clarke et al. (1997)	Yes, numerous terminals	Note: ChAT + terminals in STh are enriched with glutamate, but not GABA
	Rodent	Immunohistochemistry	Kita and Kita (2011)	Numerous fibers traversing through the nucleus	ChAT + fibers traverse from the caudodorsal to the rostroventral surface, emitting thin branches with small boutons
Nicotinic receptors	Human	Autoradiography	Pimlott et al. (2004)	Yes	NS
α3 subunit	Non-human primate	In situ hybridization	Cimino et al. (1992)	Yes, Mild	NS
α4 subunit	Human (fetal)	In situ hybridization	Agulhon et al. (1998)	Yes, moderate	NS
	Non-human primate	In situ hybridization	Quik et al. (2000)	Yes	NS
α6 subunit	Non-human primate	In situ Hybridization	Quik et al. (2000)	No	NS
α7 subunit	Non-human primate	In situ Hybridization	Quik et al. (2000)	Yes, Mild	NS
β2 subunit	Non-human primate	In situ Hybridization	Quik et al. (2000)	Uncertain	NS
β3 subunit	Non-human primate	In situ Hybridization	Quik et al. (2000)	No	NS
β4 subunit	Non-human primate	In situ Hybridization	Quik et al. (2000)	Yes	NS
Muscarinic receptors					
M2 receptors	Human	Autoradiography	Warren et al. (2007)	Yes, Mild	NS

### Noradrenergic sytem

Even though some authors evidenced projections arising from the locus coeruleus to the STh (Carpenter et al. 1981; Canteras et al. 1990), there are currently no studies available in literature addressing the noradrenergic innervation of the STh in humans. Conversely, dopamine-beta-hydroxylase (DβH) immunoreactive fibers have been evidenced in non-human primates (Ginsberg et al. 1993; Masilamoni, et al. 2017). Interestingly, MPTP-treated parkinsonian non-human primates present a significant decrease in DβH fiber density within the STh, indicating possible implications for PD (Masilamoni et al. 2017). However, the normal organization of these fibers, with particular regard to the topography of terminal arborizations, remains to be investigated.

Mild reactivity for adrenergic receptors α1 and α2 has been reported in rodents (Belujon et al. 2007). Studies addressing noradrenergic system markers are reported in Table 6.

**Table 6:** Noradrenergic system markers in the subthalamic nucleus

Noradrenergic System					
Marker	Species	Method	Author	Expression	Topography
Dopamine-β-hydroxilase (DβH)	Rodent	Immunofluorescence	Swanson et al. (1975)	No	
	Non-human primate	Immunohistochemistry	Grinsberg et al. (1993)	Yes, sparse amount of fibers coursing within the STh	
	Non-human primate	Immunohistochemistry	Masilamoni et al. (2017)	Yes, modest innervations of positive fibers	
DMI-sensitive [H]Mazindol (Noradrenaline uptake)	Rodent	Autoradiography	Javitch et al. (1985)	No	

### Purinergic receptors

Very little evidence is available on the expression and distribution of purinergic receptors in the human and non-human primate basal ganglia, despite the recent interest in the purinergic modulation of basal ganglia circuitry and the approval of the first purinergic drug for the treatment of Parkinson’s Disease in the United States and Japan. The expression of adenosine receptor A_1_ within the human STh was described by Misgeld et al. (2007), while our group has described the expression and distribution of A_2A_ receptors (Emmi et al. 2022). A_2A_ receptors were detected as dot-like reactivities colocalizing predominantly with β-III-Tubulin positive neurites, with the exception of sporadic somatic reactivites and non-β-III-tubulin positive structures, likely glial cells, as previously reported by Pelassa et al. (2019). Topographically, A_2A_R were expressed according to a dorsal to ventral decreasing gradient within the human STh.

Purinergic receptor P2X2 was investigated in rodents by Kanjhan et al. (1999) by means of in-situ hybridization and immunohistochemistry, revealing diffuse immunoreactivity of the subthalamic nucleus. In humans, the P2Y(1) receptor was found to be prominently expressed in the neurons of the subthalamic nucleus (Moore et al. 2000).

### Histaminergic system

Very little information concerning the histaminergic system in the STh is available in the literature. In rodents, marked Histamine Receptor 3 (H-3) mRNA expression was detected (Rouleau et al. 2004). In humans, binding to H-3R was investigated by means of autoradiography by Goodchild et al. (1999), but no detectable binding was found for the STh. Nevertheless, morphological investigation on histaminergic receptors is still scarce, and their expression in primates and humans remains to be confirmed.

### Opioid receptors

**Fig. 5:**
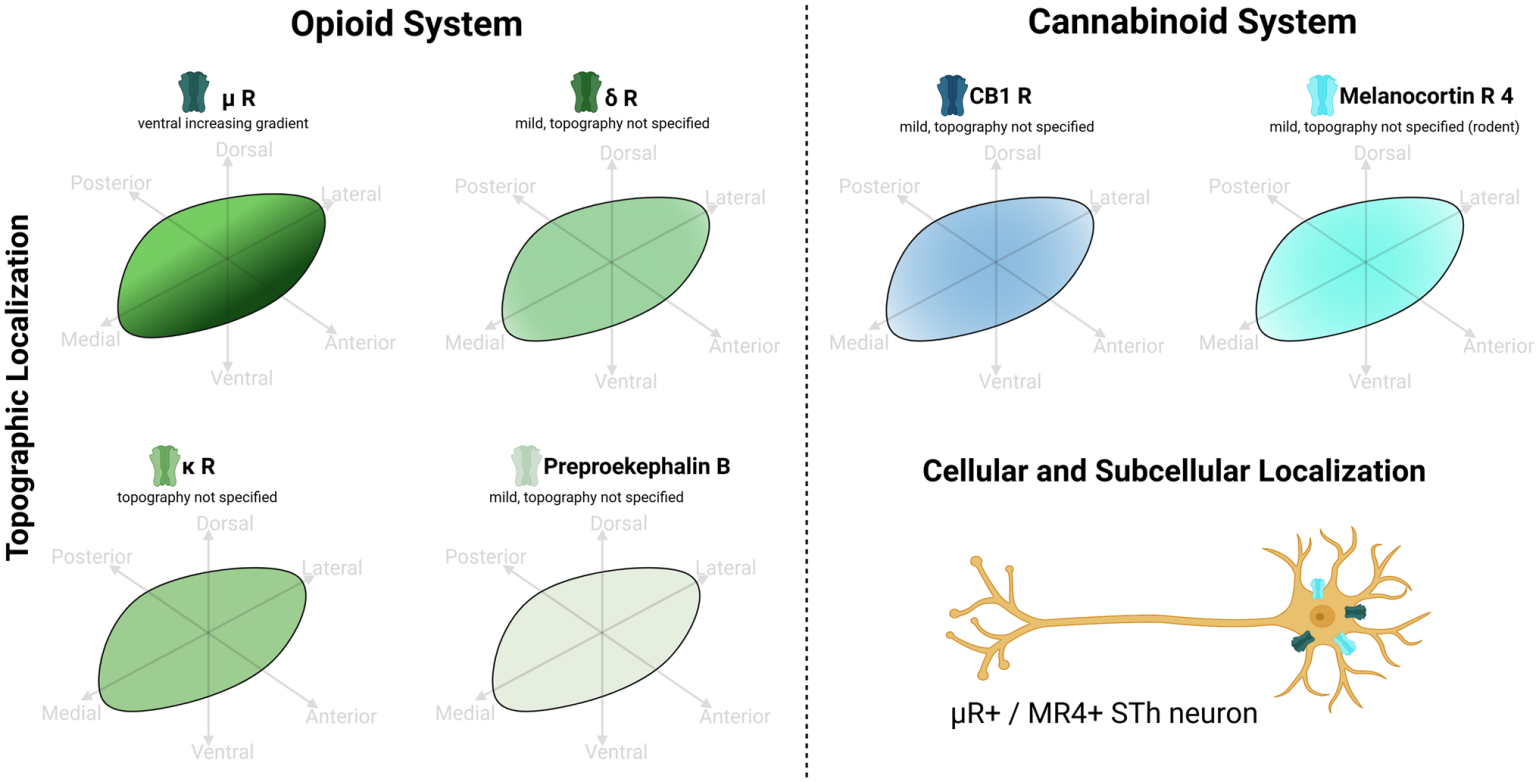
Opioid and Cannabinoid system markers in the Subthalamic Nucleus (STh). While µ receptors present a ventral increasing gradient within the STh, the topography for the major opioid and cannabinoid receptor families was not investigated. However, coexpression of µ receptor and melanocortin receptor 4 is known to occur in a set of STh neurons. Graphical representations are based on the most recent available human and non-human primate studies, as reported in Tables 7 and 8. In case of discordant findings, representation of marker topography was based on the most recent study addressing whole-volume distribution in the Subthalamic Nucleus

The expression of µ receptor in rodents was reported by Han et al. (2018), with a high rate of coexpression with melanocortin receptor 4. Non-human primate STh was found to express *µ*, *κ* and *δ* opioid receptor mRNA, with higher µ receptor expressionin the ventral STh. Preproenkephalin-b mRNA was also evidenced in primates, and its expression was found to increase after levodopa tratment in dyskinetic monkeys (Aubert et al. 2007). The presence of opioid receptor in human STh was investigated throug RNA blotting, indentifying the expression of endogenous opioid receptors (Raynor et al. 1995). mRNA of *µ* receptor was detected in human STh neurons, but no clear cellular κ and δ receptor mRNA was reported (Peckys and Landwehrmeyer 1999). Unlike primates, humans appear to present differential expression of opioid receptor mRNA. However, these findings require further confirmation via immunohistochemistry. Studies addressing opioid system markers are reported in Table 7. Figure 5 displays known topographical, cellular and subcellular localizations of opioid system markers based on the examined studies.

**Table 7:** Opioid system markers in the subthalamic nucleus

Opioid System					
Marker	Species	Method	Author	Expression	Topography (Ultrastructural and Structural)
Opioid Receptors	Human	In situ Hybdriziation	Peckys & Landwehrmeyer (1999)	High for µ, undetectable for δ and κ	Expression of µ at the level of the cellular soma. No topography reported
µ receptor	Human	RNA blotting	Raynor et al. (1995)	Yes, high expression	
	Non-human primate	In situ Hybridization, autoradiography	Aubert et al. (2007)	Yes	Higher expression in the ventral part
	Rodent	Immunofluorescence	Han et al. (2018)	Yes	NS; high coexpression with melanocortin receptor 4 expressing neurons
δ receptor	Non-human primate	In situ hybridization, autoradiography	Aubert et al. (2007)	Yes, Mild	No topography reported
κ receptor	Non-human primate	In situ hybridization, autoradiography	Aubert et al. (2007)	Yes	No topography reported
Preproenkephalin-B	Non-human primate	In situ hybridization, autoradiography	Aubert et al. (2007)	Yes, Mild	NS

### Cannabinoid receptors

Cannabinoid receptor expression was found in the rodent STh (Mailleux and Vanderhaeghen 1992) but not in non-human primates, as reported by Marani et al. (2008). Tsou et al. (1998) detected CB1 mRNA, but not CB1 receptor proteins in the rodent STh; this likely suggests CB1 receptor protein localization on terminal efferent axons. Rojo-Bustamante et al. (2018) reported the expression of CB1 in the non-human primate STh through RT-PCR analyses. Cannabinoid receptor binding in the human STh was not detected via autoradiography (Glass, Faull and Dragunow 1997). Further studies on cannabinoid receptor expression in humans are necessary to comprehend the effects of this system on STh function. Studies addressing cannabinoid system markers are reported in Table 8. Figure 5 displays known topographical, cellular and subcellular localizations of cannabinoid system markers based on the examined studies.

**Table 8:** Cannabinoid system markers in the Subthalamic Nucleus

Cannabinoid system					
Marker	Species	Method	Author	Expression	Topography
Endocannabinoid System Cannabinoid receptor 1 (CB1)	Non-human primate	Real time polymerase chain reaction (rtPCR)	Rojo-Bustamante et al. (2018)	Yes	NS
Melanocortin receptor 4	Rodent	Immunofluorescence	Han et al. (2018)	Yes	Coexpression with Mu-opioid receptors

### Calcium channels and calcium-binding proteins

While the presence of calcium channels has been reported in rodents (see Marani et al. 2008), few authors have studied the distribution of calcium channels in the human STh. Monteil et al. (2000) evidenced the presence of the α1 subunit of calcium channels within the human STh, while Yang et al. (2014) indicate that the effects of DBS may be mediated by T-type calcium channels present on subthalamic neurons.

**Fig. 6:**
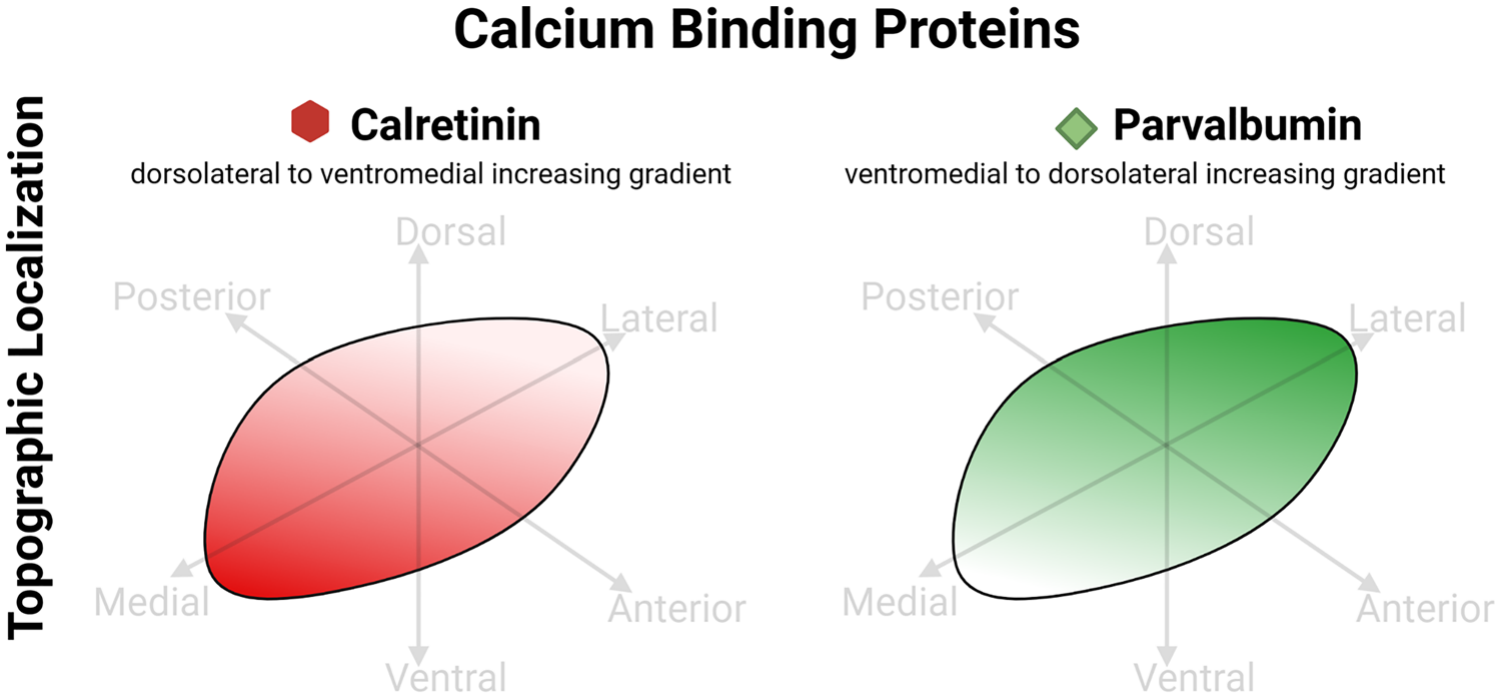
Calcium binding proteins in the Subthalamic Nucleus (STh). Calbindin is known to present an increasing dorsolateral to ventromedial gradient of expression, while Parvalbumin presents a diametrically opposite pattern, with an increasing gradient from the ventromedial to the dorsolateral pole. Graphical representations are based on the most recent available human and non-human primate studies, as reported in Table 9. In case of discordant findings, representation of marker topography was based on the most recent study addressing whole-volume distribution in the Subthalamic Nucleus

Calcium binding proteins have received substantial attention due to their involvement in calcium mediated signaling, often reflecting specific firing patterns in neurons. Studies on non-human primates have evidenced how specific calcium binding proteins, particularly calretinin, calbindin and parvalbumin can identify distinct neuronal populations within the brain, particularly the basal ganglia. Indeed, earlier evidence pointed towards calretinin as a distinct marker of non-motor STh neurons, being located predominantly at the level of the medial aspect of the structure (Fortin and Parent 1996). Marked neuronal immunoreactivity for Parvalbumin was also detected in non-human primates (Côté et al. 1991). In the human STh, a higher expression of calretinin following a dorsolateral-ventromedial increasing gradient has been evidenced, while parvalbumin is distributed according to a dorsolateral-ventromedial decreasing gradient (Morel et al. 2002; Lévesque and Parent 2005; Wu et al. 2018). Human STh neurons also displayed a moderate immunoreactivity to SMI-32 (Morel et al. 2002; Wu et al. 2018). Parent et al. (1996) and Augood et al. (1999) evidenced a specific topographic distribution of calcium-binding proteins within the human STh: in particular, calretinin was expressed mainly in neurons located in the ventromedial aspect of the nucleus, while parvalbumin was found mainly in neurons within the dorsolateral regions. However, even though a topographical organization was reported, significant overlap between territories was also evidenced. Furthermore, Alkemade et al. (2019) identified parvalbumin immunoreactivity at the level of cell bodies, as well as diffuse labeling of fibers, and calretinin labeled both cell bodies and fibers. Interestingly, Lévesque and Parent (2005) did not detect calcium binding protein expression in GAD + interneurons in the STh. Nevertheless, no studies available in literature have yet investigated the co-expression of different calcium binding proteins in human STh. While expression of parvalbumin and calretinin appears to indicate different functional gradients within the STh, boundaries between specific calcium-binding expressing territories are diffuse and do not allow for the identification of distinct STh regions. Studies addressing calcium-binding proteins and other system markers are reported in Table 9. Figure 6 displays known topographical, cellular and subcellular localizations of calcium-binding proteins based on the examined studies.

**Table 9:** Other markers and calcium binding proteins in the subthalamic nucleus

Other Markers					
Marker	Species	Method	Author	Expression	Topography
IP3 Receptor	Rodent	Immunohistochemistry	Fotuhi et al. (1993)	No	
Calcium binding proteins					
Calretinin	Human	Immunohistochemistry	Morel et al. (2002)	Yes	Diffuse; high concentrations in the ventromedial aspect of the nucleus
	Human	Immunohistochemistry	Lévesque & Parent (2005)	Yes	Increased immunoreactivity in the ventromedial aspect of the nucleus
	Human	Immunohistochemistry	Wu et al. (2018)	Yes, moderate	Increasing gradient from the dorsolateral to the ventromedial axis
	Human	Immunofluorescence, 3D Reconstruction	Alkemade et al. (2019)	Yes	Cell bodies and fibers
Parvalbumin	Human	Immunohistochemistry	Morel et al. (2002)	Yes	Diffuse; Mild concentrations in the ventromedial aspect of the nucleus
	Rodent, Non-human primates, Human	Immunohistochemistry	Hardman et al. (2002)	Yes	NS
	Human	Immunohistochemistry	Lévesque and Parent (2005	Yes	Increased immunoreactivity in the dorsolateral aspect of the nucleus
	Human	Immunohistochemistry	Wu et al. (2018)	Yes, high reactivity	Increasing gradient from the ventromedial to the dorsolateral axis
	Human	Immunofluorescence, 3D Reconstruction	Alkemade et al. (2019)	Yes	Diffuse fiber labeling and distinct neuronal labeling
SMI-32	Human	Immunohistochemistry	Wu et al. (2018)	Yes, moderate	No topographical organization

## Discussion

In the present review, we have assessed morphological studies examining the expression and distribution of markers for different neurotransmitter systems in the STh of rodents, non-human primates and humans. No studies assessed in this review have defined a clear anatomical segregation of any of the investigated neurotransmitter systems; rather, these studies have evidenced variable distribution gradients of neurochemical markers throughout the main axes of the STh, particularly in humans. This seems to suggest that complete segregation of functional territories within the STh, as originally hypothesized with the tripartite hypothesis, should be considered a conceptual simplification of a much more complex and variable internal organization of the STh (Alkemade and Forstmann 2014), with often overlapping functional territories (Emmi et al. 2020). Moreover, while the debate has previously stressed the dicotomy between functional segregation versus functional integration/convergence, revision of previous studies (Keuken et al. 2012) and recent evidence in primates (Haynes and Haber 2013; Emmi et al. 2020) indicates both functional specificity and integration. Indeed, non-human primate dendritic arborizations in the STh span across the main axis of the nucleus and occupy over two-thirds of its volume (Rafols and Fox 1976); hence, STh dendritic arborizations likely stretch across multiple functional regions, suggesting convergence between inputs from different functional areas, which appear greater than what appears based on projection patterns (Haynes and Haber 2013). Indeed, STh neurons at the center of a functional region may also receive inputs from functionally diverse cortical areas onto more distal dendrites (Bevan et al. 1997). Therefore, the output from each subthalamic neuron, although primarily driven by the cortical input matching the territory in which the neuron lies, is likely to result from the integration of functionally diverse information. This overlap between STh territories appears to increase throughout phylogenesis, with non-human primates and humans presenting little to no defined boundaries between hypothesized functional territories, possibly reflecting integration of limbic, associative and motor functions within the basal ganglia circuitry in these species (Hardman et al. 2002; Alkemade and Forstmann 2014). How this integration occurs at cellular, subcellular and molecular levels remains yet to be clarified, particularly in humans. To our knowledge, no studies in literature have yet examined the three-dimensional morphological characteristics of human STh neurons (for example, through silver impregnation techniques), and the notion of wide-spanning dendritic arborizations, likely receiving information projected to different STh regions, is mutuated from non-human primate studies (Rafols and Fox 1976) and remains to be confirmed in humans. Moreover, the expression of neurochemical markers of different neurotransmitter systems, and in particular different receptor families mediating the effects of incoming STh afferences, have not always been systematically assessed in humans. Older studies investigating neurotransmitter systems throughout the whole brain often do not consider the whole-rostro caudal extent of the STh, limiting observation to a single section of the nucleus. Moreover, unless high-magnification and thorough observation of multiple sections is performed, smaller immunoreactivities can be eaisly overlooked, as seen for dopaminergic terminal axons (Hedreen et al. 1999; François et al. 2000). This is particularly relevant for specific neurotransmitter systems, rather than others. Indeed, while glutamatergic and GABAergic systems have been more thoroughly investigated in humans, revealing a defined topographical organization mainly for GABAergic neurons and GABA receptor families in the STh, other neurotransmitters, like the dopaminergic, noradrenergic, serotoninergic and purinergic systems, have not received significant attention in humans, with at times contradicting results. Among others, dopaminergic receptors families are particularly relevant to STh function, given the known dopaminergic projections it receives from the substantia nigra (Hedreen et al. 1999; Alkemade et al. 2019; Emmi et al. 2022). We have recently evidenced how, at the level of the subthalamic nucleus, dopaminergic receptors D2 colocalize with purinergic receptors A2a, suggesting that these receptors form heteromeric receptor mosaiques (Emmi et al. 2022), and that receptor-receptor interactions occur also in the STh, as previously demonstrated for the striatum (Fernández-Dueñas et al. 2019). This notion also opens up to investigation of other receptor-receptor interactions in the STh, not only in neuronal cells, but also in glial cells; indeed, D2–A2a receptor heterodimers were discovered in the astrocytic processes of the striatum (Pelassa et al. 2019), and are potentially involved in Parkinson’s Disease pathophysiology. Similarly, we evidenced expression of A2a and D2 receptors in non-neuronal cells of the STh, even though further characterization, and more specific assays for detecting receptor-receptor interactions (i.e. proximity ligation assay, PLA), are required. Nevertheless, the possibility of investigating receptor-receptor interactions, in both neuronal and non-neuronal cells, could be of potential interest for further defining functional gradients within the STh. Indeed, while these functional gradients were traditionally defined by evaluating markers on neuronal cells and, in rare cases, their three-dimensional topography, investigating non-neuronal cells in the STh and the expression of specific glio- and neurotransmitter systems in non-neuronal cells could provide novel insights in the functional anatomy of the STh.

### Future perspectives

We believe future research on the functional anatomy of the STh should be performed by considering the most recent findings on co-occurring functional segregation and integration in the STh. Numerous older studies did not account for STh variability across the three-dimensional planes, mostly because this was beyond the scope of the study itself. STh specific studies performed on serial sections (Levesqué and Parent 2005), and the subsequent three-dimensional reconstruction of the nucleus in its whole extent (Alkemade et al. 2019; Emmi et al. 2021; 2022), are therefore highly warranted. Furthermore, we believe that, based on the available scientific literature, neurotransmitter systems and their receptors, such as the dopaminergic, serotoninergic, purinergic, and also cannabinoid and opioid systems, should receive more attention and further characterization in humans. Aside from individual receptors and their localization, the notion of receptor-receptor interactions and the detection of receptor mosaiques in the basal ganglia, greatly encourage the investigation of these phenomena also in the STh. Moreover, while research on the functional anatomy of the STh has predominantly focused on neurons, non-neuronal cells, such as astrocytes, have been characterized as potential major players in basal ganglia physiology and Parkinson’s Disease pathology; similar functional implications can be hypothesized for other nuclei, such as the STh. Hence, investigation of functional systems should also be extended to glial cells. Aside of morphological methods, other approaches have been employed to characterize the functional anatomy of the STh, with both advantages and limitations over histology. DBS itself has emerged as an intriguing tool to study functional aspects related to the STh (Aloisami et al. 2022), with the advantage of characterizing the electrophysiological properties of human neuronal populations in-vivo. Yet, this does not provide structural confirmation concerning the expression and distribution of specific neurochemical markers within the nucleus. Also, while DBS can be employed to study the electrophysiological properties of the STh, it must be noted that it is performed in humans only in case of pathology (PD, dystonia, etc.), and does not provide information concerning the physiological activity of STh neurons in this species; moreover, while electrophysiological recording of neuronal populations within the STh via DBS may suggest the involvement of specific neurotransmitters, or the expression of specific cell-membrane receptors, confirmation via morphological methods is required and should be integrated with this technique.

Lastly, while Parkinson’s Disease and other synucleinopathies generally do not affect the STh, at least via evident neuropathological alterations like Lewy bodies and neurites, functional alterations are well known to occur. The abnormal firing pattern of STh neurons in Parkinson patients is most-often regarded as a consequence of broader circuit perturbations occurring due to dopamine loss and nigral degeneration; the role of direct dopaminergic modulation, via nigro-subthalamic pathways, is often neglected (Emmi et al. 2020). Indeed, dopaminergic terminals have been detected in specific STh regions, and their loss has been reported in PD patients (Hedreen et al. 1999; François et al. 2000), likely playing a role in STh hyperactivity. While structural data suggest prominent dopaminergic innervation of non-motor areas, it is currently unknown how dopaminergic deafferentation affects functional regions of the STh, and whether or not this accounts for Parkinsonian symptoms (Antonini et al. 2023).

## Conclusions

In conclusion, available structural data concerning the functional and neurochemical anatomy of the human STh is scarce, and studies from the literature, with few exceptions, are often descriptive in nature or do not report on the distribution of the markers within the whole extent of the structure. Moreover, there are very few studies assessing the extent of functional reorganization within the STh occurring as consequence of neurodegenerative diseases, like Parkinson’s Disease.

We hope this review of literature encourages future studies on the functional and neurochemical anatomy of the human STh, and that appropriate methodological approaches will be employed to evaluate the spatial distribution of relevant markers throughout the whole nucleus.

## Data Availability

All data are available by the corresponding author upon request.
